# [^18^F]FET-PET Imaging for Treatment and Response Monitoring of Radiation Therapy in Malignant Glioma Patients – A Review

**DOI:** 10.3389/fonc.2013.00104

**Published:** 2013-04-25

**Authors:** I. Götz, A. L. Grosu

**Affiliations:** ^1^Department of Radiation Oncology, Medical Center, University FreiburgFreiburg, Germany

**Keywords:** monitoring, radiation therapy, PET-CT, glioblastoma multiforme, MRI imaging

## Abstract

In the treatment of patients suffering from malignant glioma, it is a paramount importance to deliver a high radiation dose to the tumor on the one hand and to spare organs at risk at one the other in order to achieve a sufficient tumor control and to avoid severe side effects. New radiation therapy techniques have emerged like intensity modulated radiotherapy and image guided radiotherapy that help facilitate this aim. In addition, there are advanced imaging techniques like Positron emission tomography (PET) and PET/CT which can help localize the tumor with higher sensitivity, and thus contribute to therapy planning, tumor control, and follow-up. During follow-up care, it is crucial to differentiate between recurrence and treatment-associated, unspecific lesions, like radiation necrosis. Here, too, PET/CT can facilitate in differentiating tumor relapse from unspecific changes. This review article will discuss therapy response criteria according to the current imaging methods like Magnet resonance imaging, CT, and PET/CT. It will focus on the significance of PET in the clinical management for treatment and follow-up.

## Tracers for Brain Tumors

Positron emission tomography (PET) is a functional imaging method that has gained widespread use in the assessment of brain tumors. PET-tracers currently used for imaging of brain tumors are mostly radio labeled amino acid (AA) tracers. These AA are preferentially taken up by tumor cells (Derlon et al., 1989; Heiss et al., 1999; Grosu et al., 2011) due to an overexpression of amino acid transporters, while the uptake of the normal brain tissue is relatively low. It has been demonstrated that AA uptake in tumor tissue is almost entirely mediated by type l-AA carriers (Heiss et al., 1999). It has been suggested in a rat model that brain tumors can stimulate transporter expression, especially in their vasculature (Miyagawa et al., 1998). The most common tracers for malignant brain tumors are ^18^Fluoro-*O*-(2) fluoroethyl-l-tyrosine ([^18^F]FET) and [^11^C] Methionine (MET).

[^11^C] Methionine (MET) is a physiologic amino acid labeled with a carbon-11 isotope, which has a half-life time of 20 min. Its uptake correlates with cell proliferation *in vitro*, Ki-67 expression, nuclear antigen expression, and microvessel density in proliferating cells (Dhermain et al., 2010). First studies on AA tracers were made with MET. ^18^Fluoro-*O*-(2) fluoroethyl-l-tyrosine ([^18^F]FET) is an amino acid labeled with fluorine-18 which has a half-life of 110 min.

Due to the short half-life of a positron-emitting radioisotope like carbon-11, the radiotracers labeled with carbon-11 require a cyclotron in close proximity to the PET imaging facility. The half-life of fluorine-18 is long enough that radiotracers labeled with fluorine-18 can be manufactured commercially at off-site locations and shipped to *outlying facilities*.

In clinical practice, FET and MET have been shown to be equally sensitive and specific (Weber et al., 2000; Astner et al., 2005; Grosu et al., 2011). In case of low-grade glioma FET can also reveal hot spots and suspected regions of histological upgrading of the tumor (Popperl et al., 2007).

[^18^F]2′-fluoro*-*2′*-*deoxyglucose (FDG), an analog of glucose that is labeled with fluorine-18, is often used in extracerebral tumors. FDG shows a high uptake in gray matter, resulting in a poor tumor to background ratio, especially in low-grade glioma. Thus, FDG is currently restricted to special situation like cerebral lymphomas (Hoffman et al., 1993), where it is of prognostic value (Kasenda et al., 2013).

^68^Ga-DOTATOC (DOTA^0^-Phe^1^-Tyr^3^-octreotide) or other somatostatin analogs are very sensitive in the detection and delineation of meningioma and its possible infiltration in sagittal sinus or falx (Milker-Zabel et al., 2006).

^18^F-DOPA, a l-3,4-dihydroxyphenylalanine labeled with fluorine-18 shows an increased uptake in malignant glioma and have been shown to be comparable to MET (Becherer et al., 2003).

[^18^F]3′-deoxy-3′-fluorothymidine (FLT), a nucleoside, shows an increased expression of thymidine kinase and cell proliferation (Ullrich et al., 2008) and correlates with Ki-67. It allows a non-invasive assessment of tumor proliferation as well as early response to chemotherapy by PET (Jacobs et al., 2005). Since ^18^F-FLT does not cross intact brain-blood-barrier (BBB) it does not show uptake in low-grade tumors or stable lesions but ^18^F-FLT visualize high-grade (grade III or IV) tumors with a disrupted BBB (Chen et al., 2005).

^18^F-fluoromisonidazole (^18^F-FMISO) has the ability to visualize the hypoxic cell fraction of tissue (Cher et al., 2006) and makes it possible to achieve escalation of the radiation dose at these crucial points. However, at this point, FLT and FMISO are not yet well established in the clinical management.

## Therapy of Malignant Glioma

Standard treatment for malignant glioma is based on surgery followed by combined radio-chemo therapy up to 60 Gy and adjuvant chemotherapy with temozolomide (Stupp et al., 2005). Nevertheless, there are additional therapy approaches with radioactive seeds, other chemotherapy agents like irinotecan or antiangiogenic agents like bevacizumab.

Frequently used radiation therapy (RT) techniques in patient with malignant glioma are: 3-D conformal RT and, especially in cases of re-irradiation, stereotactic fractioned RT. Furthermore, intensity modulated RT, rapid arc techniques and image guided RT are also frequently used (Narayana et al., 2006; Hermanto et al., 2007).

## Response Monitoring

During and after treatment, therapy response should be evaluated. Currently, most protocols use conventional imaging techniques like CT and Magnet resonance imaging (MRI) for this purpose. It is, however, important to differentiate between regression, treatment related changes and true recurrence. In this situation, AA-PET has become of great value due to its superior sensitivity for vital tumor tissue (Popperl et al., 2005).

## Limitations of MRI

Magnet resonance imaging with its high spatial resolution is an inexpendable tool in diagnosis, RT planning, and follow-up in patients with malignant glioma. However, there are pitfalls since this imaging method is not tumor specific (Table 1).

**Table 1:** **Studies showing limitation of conventional MRI**.

Author	Evidence	Reference
Taal	18 out of 36 patients, (50%) were diagnosed with pseudoprogression 4 weeks after radiation therapy and concomitant temozolomide.	Taal et al. (2008)
Mullins	Individual patterns of enhancement are not enough to distinguish necrosis from predominant tumor progression.	Mullins et al. (2005)
Rachinger	For patients with glioma undergoing multimodal treatment or various forms of irradiation, conventional follow-up with MRI is insufficient to distinguish between benign side effects of therapy and tumor recurrence	Rachinger et al. (2005)

Diagnostic criteria in routine MRI tumor imaging are generally based on the extent of contrast enhancement, which is caused by a breakdown of the BBB (Wen et al., 2010). However, a disruption of the BBB can also occur as a result of recent surgery or RT. On the other side, there can be tumor parts where the BBB is not yet affected. Furthermore, the contrast enhancement is in many cases smaller than the real tumor dimension, leading to an underestimate of tumor dimensions. This phenomenon is very common in low-grade glioma.

In addition, new emerging therapies like VEGF-inhibitors can reduce disturbance of the BBB, causing a decrease of contrast enhancement on MRI without an influence of the tumor dimension, which is referred to as “pseudo-regression.” It is also seen after application of corticosteroids, which can also reduce leakage of blood vessels (Jacobs et al., 2005).

After RT, brain lesions may remain avid to contrast agents like Gadolinium but may become negative on AA-PET, which is indicative of good local tumor control. For example, if post-treatment changes like radio necrosis occurs, it is frequently associated with an increase of contrast enhancement which can mimic tumor progression (Giglio and Gilbert, 2003).

Such phenomenon occurring after treatment are called pseudoprogression and pseudoresponse (Brandsma and van den Bent, 2009). They point at a problematic discrepancy between morphological MRI imaging and true tumor behavior.

In case of pseudoprogression, increase of the contrast enhancement on MRI is not associated with real tumor growth (Taal et al., 2008). In such situations, AA-PET as a functioning imaging modality can help differentiate between real tumor progress and treatment related changes. Table 2 gives an overview of studies evaluating PET in the follow-up of glioma and its abilities to differentiate between recurrence of tumor, pseudoprogression, and radiation necrosis.

**Table 2:** **Studies evaluating the role of AA-PET in the follow-up of glioma and in the differentiation between recurrent tumors and pseudoprogression/radiation necrosis**.

Author	Title	Reference
Tripathi M	Comparison of F-18 FDG and C-11 methionine PET/CT for the evaluation of recurrent primary brain tumors	Tripathi et al. (2012)
Langleben, D.	PET in Differentiation of recurrent brain tumor from radiation Injury	Langleben and Segall (2000)
Pöpperl, G.	Value of O-(2-[18 F] fluoroethyl)-l-tyrosine PET for the diagnosis of recurrent glioma	Tripathi et al. (2012)
Hein, P. A.	Diffusion-weighted imaging in the follow-up of treated high-grade glioma: tumor recurrence versus radiation injury	Hein et al. (2004)
Terakawa, Y.	Diagnostic accuracy of 11C-methionine PET for differentiation of recurrent brain tumors from radiation necrosis after radiotherapy	Terakawa et al. (2008)
Grosu, A. L.	l-(methyl-11C) methionine positron emission tomography for target delineation in resected high-grade gliomas before radiotherapy.	Grosu et al. (2005a)
Tsuyunguchi, N.	Methionine positron emission tomography for differentiation of recurrent brain tumor and radiation necrosis after stereotactic radiosurgery in malignant glioma	Tsuyuguchi et al. (2004)
Dhermain, F. G.	Advanced MRI and PET imaging for assessment of treatment response in patients with gliomas	Dhermain et al. (2010)

Sophisticated MR techniques, like MR spectroscopy and perfusion (Rock et al., 2004) are not standardized until now and their reproducibility between different facilities is difficult. In addition, MRI spectroscopy has multiple methodological limitations that impede its use in clinical practice; these limitations are, however, beyond the scope of this article.

## Advantages of Amino Acid – PET

AA-PET (FET and MET) is often used when a recurrence of the tumor is assumed on MRI after treatment. In this situation AA-PET has been shown to be superior to MRI regarding discrimination of true tumor growth from treatment related changes. Reported sensitivity rates of AA-PET range from 75 to 100% and specificity from 60 to 100% (Pauleit et al., 2005; Tripathi et al., 2012), respectively. It helps to confirm the diagnosis of recurrence and is used for treatment planning in case of re-irradiation, as it enables better tumor delineation.

^18^F-FET has been also shown to predict treatment response after radiotherapy. PET-responders showed a significant longer overall survival than non-responders (Piroth et al., 2011). Reduced AA uptake in brain tumors under therapy correlates with treatment response (Nariai et al., 2005).

It was shown that the target volume delineation for re-irradiation according to MRI can vary extremely (Grosu et al., 2005a). In this situation an AA-PET should be used. Target volume delineation according to AA-PET is much more reliable and less variable between different observers (Grosu et al., 2005a).

Current European guidelines have determined cut-off values for semiquantitative PET analyses (SUV of tumor compared to SUV of healthy brain tissue) to be applied in various clinical settings depending on the tracer that is used (Vander Borght et al., 2006). For example, the current cut-off value of the tumor to background uptake-ratio for differentiating neoplastic brain tissue from healthy surrounding tissue with [^18^F]FET has been set at 1.6. This makes it especially useful for integration into RT planning, since it enables semi-automated tumor delineation based on threshold values for FET-uptake and sets a vantage point for prospective studies.

Consequently, preliminary studies show a significant longer overall survival in patients who were irradiated on the basis of PET or SPECT in contrast to patients who were treated on the basis of MRI-based RT planning alone (Grosu et al., 2005b). Further studies are needed, but these data suggest that AA-PET might contribute largely to improvement of patient care.

## Limitation of PET

Numerous disadvantages of PET must be taken into account: while the method itself is not associated with adverse effects due to the use of only trace amounts of physiologic amino acids, it leads to a certain radiation exposure to the patient. However, radiation dosage received from modern PET imaging (ranging from 2 to 6 mSv), is negligible against the background of the RT dose delivered for the treatment and the poor prognosis of the disease at hand.

Furthermore, low spatial resolution of PET-studies is a limitation. Current scanners achieve about 4–8 mm compared to 1 mm on MRI. This can lead to false negative findings as PET might not detect very small lesions. On the other hand, the clinical relevance of lesions smaller than 5 mm remain debatable.

Positron emission tomography-tracers labeled with carbon-11 have a short half-life and a cyclotron is needed for its manufacture. With the introduction of 18F-fluorine marked tracers, this disadvantage has, however, been eliminated.

Expertise is needed for interpretation of PET data. The diagnostic value of amino acid uptake in brain tissue depends on multiple factors: the tumor to brain ratio for system L transport substrates can be relatively low, like mentioned above (Vander Borght et al., 2006). Unspecific uptake is possible shortly after operation or biopsy. Hence, due to the functional and not anatomical nature of PET-studies, specificity is generally low as PET does not allow to differentiate pathologic amino acid uptake (tumor) from unspecific uptake. For example [^18^F]FET signal is physiologically increased in vascular malformations or the venous sinuses, which can mimic tumor extension to the contralateral hemisphere in the case of sinus sphenoidalis. [^11^C]MET signal is increased, e.g., in the lacrimal gland. Thus, MRT imaging will remain inexpendable for tumor diagnostics and interpretation of AA-PET.

Much hope lies therefore in the upcoming of combined MR/PET scanners that will allow for superior diagnostic power in only one study, minimizing time consumption, and patient discomfort (Judenhofer et al., 2008).

## Summary

In brain tumors, PET is currently recommended when MRI is inconclusive especially in the setting of post-therapeutic care. PET shows a greater specificity in differentiation between tumor and post-treatment changes, while MRI is inexpendable in the evaluation of morphological features.

Positron emission tomography has become an important imaging technique to improve the definition of the target volume for irradiation and to decrease the inter-observer variability (Grosu et al., 2005a). There is also evidence that using PET for RT planning improves overall survival (Grosu et al., 2005b). This has yet to be evaluated in larger randomized studies.

Positron emission tomography is more and more established as imaging tool in assessment of treatment response, recurrence, and follow-up of malignant glioma patients.

Combination of PET and MRI imaging into one study (PET/MRI) could further improve patient care and facilitate therapy management.

## Conflict of Interest Statement

The authors declare that the research was conducted in the absence of any commercial or financial relationships that could be construed as a potential conflict of interest.
